# Selective binding modes and allosteric inhibitory effects of lupane triterpenes on protein tyrosine phosphatase 1B

**DOI:** 10.1038/srep20766

**Published:** 2016-02-11

**Authors:** Tiantian Jin, Haibo Yu, Xu-Feng Huang

**Affiliations:** 1 Centre for Translational Neuroscience, School of Medicine, University of Wollongong, and Illawarra Health and Medical Research Institute (IHMRI), Wollongong, NSW 2522, Australia; 2 School of Chemistry, University of Wollongong, Wollongong, NSW 2522, Australia

## Abstract

Protein Tyrosine Phosphatase 1B (PTP1B) has been recognized as a promising therapeutic target for treating obesity, diabetes, and certain cancers for over a decade. Previous drug design has focused on inhibitors targeting the active site of PTP1B. However, this has not been successful because the active site is positively charged and conserved among the protein tyrosine phosphatases. Therefore, it is important to develop PTP1B inhibitors with alternative inhibitory strategies. Using computational studies including molecular docking, molecular dynamics simulations, and binding free energy calculations, we found that lupane triterpenes selectively inhibited PTP1B by targeting its more hydrophobic and less conserved allosteric site. These findings were verified using two enzymatic assays. Furthermore, the cell culture studies showed that lupeol and betulinic acid inhibited the PTP1B activity stimulated by TNFα in neurons. Our study indicates that lupane triterpenes are selective PTP1B allosteric inhibitors with significant potential for treating those diseases with elevated PTP1B activity.

Protein Tyrosine Phosphatase 1B (PTP1B) is an intracellular protein which is widely expressed in the body including the brain, liver, muscles, and adipose tissue, and which is up-regulated in obesity, type 2 diabetes and breast cancer^1^,^2^,^3^. Obesity is a major health problem leading to various life-threatening diseases such as diabetes, cardiovascular disease and certain cancers^4^. An elevated PTP1B level contributes to the development of obesity and its related metabolic disorders^5^,^6^. Considerable efforts have been made towards new anti-obesity drug developments. PTP1B has been considered as a therapeutic target for treating obesity. Previous studies have shown that inhibiting neuronal PTP1B in obese mice reduces fat deposition, improves energy expenditure and prevents weight gain^7^,^8^.

However, there are some outstanding challenges in PTP1B-based small-molecule therapeutics. First, it is difficult to achieve inhibition selectivity against PTP1B by targeting the active site. PTP1B is a member of the Protein Tyrosine Phosphatase (PTP) family, which contains more than 100 members. Most PTPs have a consensus active loop signature (H/V)C(X)_5_R(S/T), where the cysteine (C) is a conserved active site that is essential for enzyme catalysis^9^. Of particular interest, PTP1B shares a 74% identical sequence in its catalytic domain with T-cell protein tyrosine phosphatase (TCPTP)^10^ and they have almost superimposable active sites. TCPTP has different biological functions and signalling pathways from PTP1B as demonstrated in mouse models^11^. Studies have shown the regulatory functions of TCPTP on the immune system^12^. Homozygous TCPTP-deficient mice died at 3–5 weeks of age due to the haematopoietic defect caused by immune system damage^11^. Therefore, an effective PTP1B inhibitor needs to have sufficient selectivity for PTP1B over TCPTP. Second, inhibitors targeting the intracellular target PTP1B need to have satisfactory cellular penetration. Current PTP1B inhibitors are designed to bind to the PTP1B active site, the phosphotyrosine (pTyr)-binding pocket, serving as competitive inhibitors to reduce PTP1B activity^13^. These PTP1B inhibitors mimic pTyr and are negatively charged at a physiological pH. Consequently, it is difficult for most PTP1B inhibitors to penetrate the cell membrane^14^.

Due to the challenges of the active site targeted inhibitors mentioned above, an alternative drug design strategy has been proposed to develop inhibitors targeting the PTP1B allosteric site instead^13^. Recent X-ray crystallographic studies have revealed an allosteric transition in PTP1B accompanying its catalysis, which is situated about 20 Å away from the catalytic domain including active site Cys215 and catalytic loop consisting His214, Ser216, Ala217, Gly218, Ile219, Gly220 and Arg221^15^,^16^ (Fig. 1a,b). The catalytic WPD loop (Trp179, Pro180, and Asp181) and neighbouring residues can exist in two distinct conformations: “open” and “closed”^17^ (Fig. 1c). In the open state, the WPD loop stands beside the active site to form an open binding site, which is accessible for substrates. In contrast, in the closed state, the WPD loop closes over the binding site, forming a catalytically competent state. Thus an allosteric inhibitor can be designed to prevent the movement of the WPD loop and maintain the WPD loop in an open (inactive state)^16^. Unlike the active site of PTP1B, the allosteric site is not well conserved among PTPs and is substantially less polar^15^. Thus targeting the allosteric site might offer a promising approach to developing PTP1B inhibitors with both improved selectivity and bioavailability. The high-resolution X-ray structures of PTP1B, in complex with three allosteric inhibitors, including compound 2 and compound 3 (Fig. 2), show that these inhibitors target the allosteric site formed by α3, α6 and α7^16^. Encouragingly, these allosteric inhibitors show high potency in inhibiting PTP1B with selectivity over other PTPs^16^. Considering the limited selective PTP1B inhibitors on trial^18^, allosteric inhibition becomes a promising strategy to discover selective PTP1B inhibitors^19^,^20^.

Recently, the lupane triterpenes, lupeol, lupenone, betulin and betulinic acid (Fig. 2), have been shown to be potent PTP1B inhibitors *in vitro*^21^,^22^,^23^. Na *et al.* showed that lupeol can inhibit PTP1B with a high potency (IC_50_ = 5.6 μM), and acts as a non-competitive inhibitor of PTP1B^21^. Therefore lupeol might target a binding site other than the active site of PTP1B. We propose the lupane triterpenes as potential PTP1B allosteric inhibitors and aim to reveal their molecular inhibitory mechanisms. First, we performed molecular docking, molecular dynamics simulations and binding affinity calculations to predict the allosteric binding site targeted by lupane triterpenes and characterise the key interactions and residues involved in the binding. Second, we used enzymatic assays to determine the inhibition selectivity of lupane triterpenes against PTP1B over TCPTP. We carried out kinetic assays to confirm their allosteric binding modes. Finally, we examined the cellular activities of selected lupane triterpenes for their ability to inhibit PTP1B activity in hypothalamic neurons. This combination of molecular modelling, enzymatic assays and cell culture studies has established lupane triterpenes as potent and selective PTP1B inhibitors with significant potential for treating diseases with elevated PTP1B activity.

## Methods

### Homology modelling of PTP1B

The PTP1B crystal structure (PDB id: 1T49) used in this study was the open, inactive conformation containing 282 amino acids, in which the α7 was not resolved (labelled as PTP1B282). Considering that there was no crystal structure of PTP1B with α7 available in the inactive state, and also the important role of α7 in allosteric inhibition, homology modelling with Modeller 9v8 was carried out to construct the missing helix based on the active conformation of PTP1B (PDB id: 1PTY) (labelled as PTP1B299)^24^. 100 models were generated and the best model based on the DOPE energy was chosen and subjected to equilibrium molecular dynamics simulations (40 ns) to equilibrate and optimise the modelled structure (see Molecular dynamics simulations).

### Molecular docking

Docking simulations were carried out with Autodock Vina (version 1.1.2)^25^. The docking protocol was established by re-docking compound 2 to PTP1B282 as in the co-crystal structure^16^. The protonation states of the titratable groups in PTP1B were assigned at pH 7.0 by PROPKA3.1^26^. Docking was performed sequentially in two steps. Initially, a box large enough to cover the whole protein (75 Å × 60 Å × 60 Å) was used to detect potential binding pockets. Then, the second step involved localised docking with a smaller box (22.5 Å × 22.5 Å × 22.5 Å) centred at the potential binding site of interest (e.g. the active site, the allosteric site, or other site of interest identified from the blind docking procedure). In each focused docking study of lupane triterpenes targeting PTP1B282 or PTP1B299, 20 conformations of the complexes with the lowest binding affinities were listed for further analysis and the best binding mode was selected to be the initial structure for the molecular dynamics simulations. Multiple dockings, based on different snapshots sampled from equilibrium molecular dynamics simulations (PTP1B282 and PTP1B299), were performed. Qualitatively, the identified populated binding poses are similar.

### Molecular dynamics simulations

Molecular dynamics simulations were performed to study the stability and flexibility of the PTP1B-lupane triterpenes complexes. 9 different systems were set up (Table S1). All of the systems were solvated in a box of TIP3P water molecules, which extended about 12 Å from the surface of the protein, and the systems were neutralised with counter ions of Na^+^. The salt (NaCl) concentration was set to 0.15 mol/L. Simulations were carried out using NAMD 2.9^27^. The protein and the ligands were represented with the non-polarisable CHARMM PARAM27 force field^28^ and the GAFF force field^29^, respectively. All of the systems were simulated in periodic boundary conditions using the Langevin algorithm to maintain the temperature at 298.15 K, and the Langevin Piston Nose-Hoover method to keep the pressure constant at 1.0 bar. The electrostatic interactions were calculated using the Particle Mesh Ewald (PME) method^30^. The van der Waal forces were treated with a cut-off of 12 Å. All of the covalent bonds involving hydrogen were kept rigid using the Rattle algorithm and time step set to 1.0 fs. In the equilibrium simulations of the homology modelling of PTP1B, a harmonic restraint on the backbone atoms except α7 was applied with a decreasing force constant from 64.0 to 1.0 kcal/mol/Å^2^ over 10 ns. The simulations were continued for 30 ns without any restraints. For the molecular dynamics simulations of the docked complexes, a harmonic restraint on the backbone atoms was applied with a decreasing force constant from 32.0 to 1.0 kcal/mol/Å^2^ over 3 ns followed by 20 or 100 ns equilibrium simulations (Table S1).

### Binding free energy calculation

Free energy perturbation distributed replica-exchange molecular dynamics (FEP/λ-REMD) was applied to calculate binding free energy^31^,^32^. The motivation to carry out computationally intensive absolute binding free energies is because the binding modes for lupeol and betulinic acid predicted by docking and subsequently molecular dynamics simulations are rather different. This could be difficult for relative binding free energy calculations, which assume that their available conformational spaces are significantly overlapped^33^. To be consistent with previous literature, we call the free energy difference between the unbound ligand in the aqueous solution and the bound ligand in the binding pocket of the target protein as the absolute binding free energies. The total free energy was decomposed into four terms, namely repulsive and dispersive components of the Lennard-Jones potential according to the Weeks-Chandler-Anderson scheme, the electrostatic contribution, and the restraining potential. The ligand was decoupled from the environment in four steps via four thermodynamic coupling parameters (λ). No corrections have been applied to the potential electrostatic finite-size artefacts^34^,^35^. In order to speed up the convergence, each λ-staging FEP window was treated as a replica and the λ-exchange occurred along the entire alchemical reaction path. This approach has been successfully used to study the interactions between glycoside hydrolases and polysaccharides^36^. Generally, for both PTP1B-lupeol and the PTP1B-betulinic acid complex, the FEP/λ-REMD simulations started from a 100 ns equilibrated snapshot. A set of 64 replicas (36 repulsive, 12 dispersive, and 16 electrostatic) was applied in the simulations with an exchange frequency of one every 1,000 steps (1 ps). For each replica, a total of 1.0 ns simulations were carried out in which the last 0.8 ns simulations were averaged to determine the ligand binding free energies. The protein-ligand complexes included a positional translational restraint with a force constant of 10.0 kcal/mol/Å^2^. The error analyses were estimated using four sequential simulations of 0.2 ns each.

### Analysis

Trajectory snapshots were saved every picosecond (i.e. every 1,000 steps) and analysed using either the the molecular visualisation program VMD 1.9^37^ or CHARMM^38^.

### Inhibiting TCPTP by *p*NPP phosphatase assay

The inhibition of TCPTP was determined by using the EnzoLyte Colorimetric *p*NPP Protein Phosphatase Assay kit (Anaspec, San Jose, CA). The recombinant human TCPTP protein (ab42575) was purchased from Abcam Inc (Cambridge, MA). Lupeol, betulin, and betulinic acid were purchased from Sigma-Aldrich (Castle Hill, NSW). Lupenone was purchased from Faces Biochemical Co Ltd (Wuhan, P R China). Briefly, TCPTP was incubated in different concentrations of compounds for 10 min in assay buffer at 25 °C, and then the reaction was initiated by adding the *p*NPP reagent and stopped by NaOH after 30 min at 25 °C. All readings were calibrated on the negative control wells without enzymes. The TCPTP inhibition potency of the lupane triterpenes was determined by IC_50_, calculated using the GraphPad Prism 5 program (GraphPad Software Inc, La Jolla, CA). In addition, both the inhibition of TCPTP and PTP1B (ab42572, Abcam Inc, Cambridge, MA) were performed with the control drug compound 3 (Merck, Frenchs Forest, NSW), a reported allosteric PTP1B inhibitor. The PTP1B assay process was the same as the TCPTP assay mentioned above.

### PTP1B inhibition kinetics assay

The PTP1B-catalysed hydrolyses in the presence of betulin and betulinic acid were assayed at 25 °C, and the *p*NPP Protein Phosphatase Assay kit is described above. The reaction mixture consisted of six different concentrations of *p*NPP (1.0, 2.0, 4.0, 8.0, 16.0, and 20.0 mM) and was used as a PTP1B substrate with different concentrations of lupeol, betulin, and betulinic acid. The Lupeol group was used as a control group. The absorbance at 405 nm was detected by a spectraMAX 384 microplate spectrophotometer twice every minute for a total of 15 min. Michaelis-Menten constant (*K*_m_) and maximum velocity (*V*_max_) of PTP1B were determined via Lineweaver-Burk plots using the GraphPad Prism 5 program (GraphPad Software Inc, La Jolla, CA).

### Cell culture and reagents

Mouse hypothalamic cell line (mHypoE-46) neurons were grown in monolayer in Dulbecco’s modified Eagle medium (DMEM) (Sigma D5796, Castle Hill, NSW) with 10% fetal bovine serum (FBS) (SAFC Biosciences Inc, Lenexa, KS) and 1% penicillin/streptomycin. They were maintained at 37 °C with 5% CO_2_. Prior to treatment, the cell culture medium was replaced with DMEM containing 1% penicillin/streptomycin for 4 h. Murine TNFα was obtained from Sigma-Aldrich (Castle Hill, NSW). Lupeol and betulinic acid were dissolved in Dimethyl sulfoxide (DMSO), then diluted in sterile water, and mixed with serum free cell culture medium. The DMSO concentration was controlled below 0.15%. The final concentrations of lupeol and betulinic acid in the cell culture medium were 28 μM (5× of the IC_50_ value) and 7.5 μM (5× of the IC_50_ value), respectively.

### Western blot analysis

The method was described in our previous study with modifications^39^, mHypoE-46 neurons were washed with ice-cold PBS and lysed in NP-40 lysis buffer (Invitrogen Australia Pty Ltd, Mulgrave, VIC) containing a protease inhibitor cocktail, beta-glycerophosphate and phenylmethanesulfonyl fluoride (PMSF) (Sigma, Castle Hill, NSW). Cell lysates were centrifuged at 14000 rpm for 10 min at 4 °C. The supernatants were collected and protein concentrations were determined using the DC assay (Bio-Rad Laboratories, Gladesville, NSW) according to the manufacturer’s instructions. Equal amounts of protein (10 μg) were separated on 4–12% Bis-Tris gels (Bio-Rad Laboratories, Gladesville, NSW,) using SDS-PAGE. Following electrophoresis (120 V for 2 h), the proteins were transferred to polyvinylidene difluoride membranes (100 V for 1 h). Membranes were blocked in 5% bovine serum albumin (BSA), followed by incubation with the primary antibodies PTP1B (sc-1718, 1:500, Santa Cruz Biotechnology Inc, Dallas, TX) in 1% BSA overnight at 4 °C. Following washes (3 × 5 min) in Tris Buffered Saline +0.1% Tween 20 (TBST), membranes were incubated with horseradish peroxidase conjugated secondary antibodies for 1 h at 25 °C. Blots were visualised using enhanced chemiluminescence (ECL) detection reagents (Ge Healthcare, Rydalmere, NSW). The bands corresponding to the proteins of interest were scanned and densitometrically analysed using the automatic imaging analysis system Quantity One (Bio-Rad Laboratories, Gladesville, NSW). All quantitative analyses were normalised to β-actin.

### Immunoprecipitation assay of PTP1B

Based on the instructions of the Pierce Classic IP Kit (#26146, Thermo Fisher Scientific Inc, IL) with minor modifications^40^, PTP1B was immunoprecipitated using the 25 μg PTP1B antibody (sc-1718, Santa Cruz Biotechnology Inc, Dallas, TX) for 12 h at 4 °C. Immune complexes were isolated by adding the protein G-agarose, washed three times in the phosphatase assay buffer from the EnzoLyte Colorimetric *p*NPP Protein Phosphatase Assay kit (Anaspec, San Jose, CA), and resuspended in the same buffer. Samples were analysed for the phosphatase activity using the same *p*NPP assay kit mentioned above.

### Statistical analysis

Data from the western blot analysis and immunoprecipitation assays were analyzed using the SPSS 19 statistical package (SPSS, Chicago, IL). One-way analysis of variance (ANOVA) was applied followed by the *post-hoc* Tukey-Kramer honestly significant difference (HSD) test.

## Results

### Lupeol binds to PTP1B in the allosteric site

**Figure 2:**
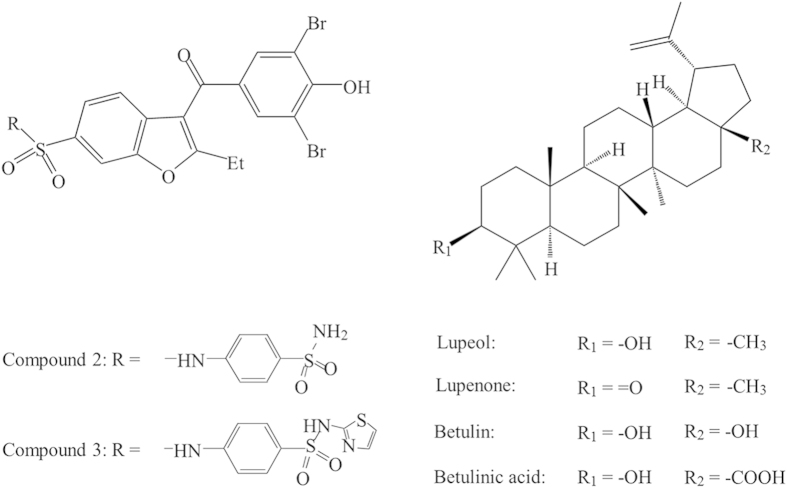
The formula of allosteric ligands used. Compounds 2 and 3 have been reported to be allosteric inhibitors by Wiesmann *et al.*^16^. Four members of the lupane triterpenes are selected for this work.

**Figure 3:**
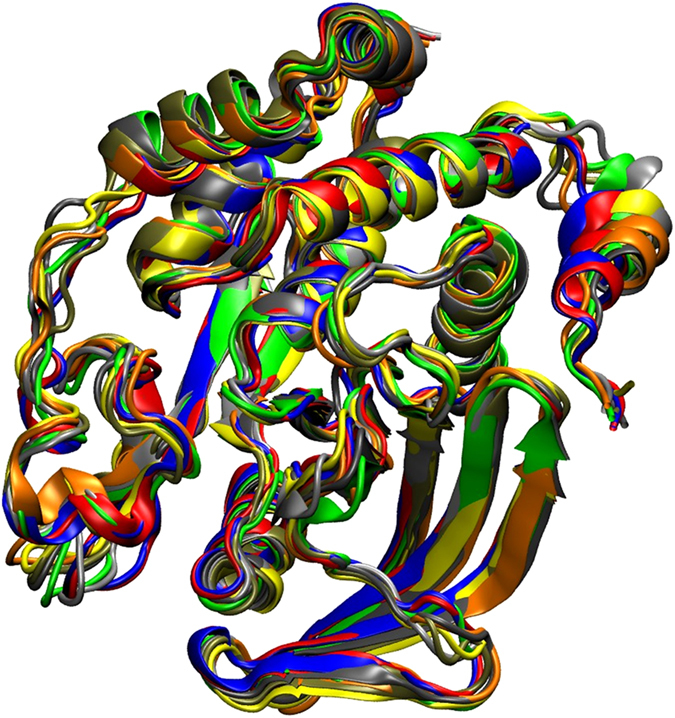
Equilibrated structures of the homology model for PTP1B299. The snapshots sampled from the molecular dynamics simulations at 10 ns (red), 15 ns (grey), 20 ns (orange), 25 ns (yellow), 30 ns (tan), 35 ns (silver), and 40 ns (green), are superimposed with the homology model using Modeller (blue).

The homology model of PTP1B in the inactive state with compound 2 (PTP1B299 w/Compound 2, see Fig. 2) was simulated for 50 ns: the first 10 ns with a harmonic restraint on the backbone atoms of the resolved crystal structure while optimising the modelled α7; and the remaining 40 ns being free molecular dynamics simulations. The snapshots sampled during the simulations are shown in Fig. 3. In general, the tertiary structures were stable for over 40 ns with the modelled α7 having greater flexibility. As expected for the relative short timescale of the free molecular dynamics simulations, no closure of the active site was observed and PTP1B remained in the inactive state.

One of the remaining problems of allosteric inhibition is to characterise the binding modes of these inhibitors and identify the key residues involved in the binding. In particular, the truncated form of PTP1B without α7 is four times less active, indicating the importance of α7 in PTP1B inhibition^16^. In addition, X-ray structures have revealed that in the presence of an allosteric inhibitor, α7 is disordered and thus the molecular details of α7 in allosteric inhibition remain unclear^16^. To determine the role of α7, molecular docking studies (blind and focused docking) were performed for PTP1B in the presence of α7 (PTP1B299) or absence of α7 (PTP1B282). These docking studies revealed similar binding modes for lupeol with the interactions for PTP1B299 being stronger. The following discussions focus mainly on PTP1B299 and note the differences between PTP1B282 and PTP1B299.

### α7 is involved in forming the allosteric binding site in PTP1B299

**Figure 4:**
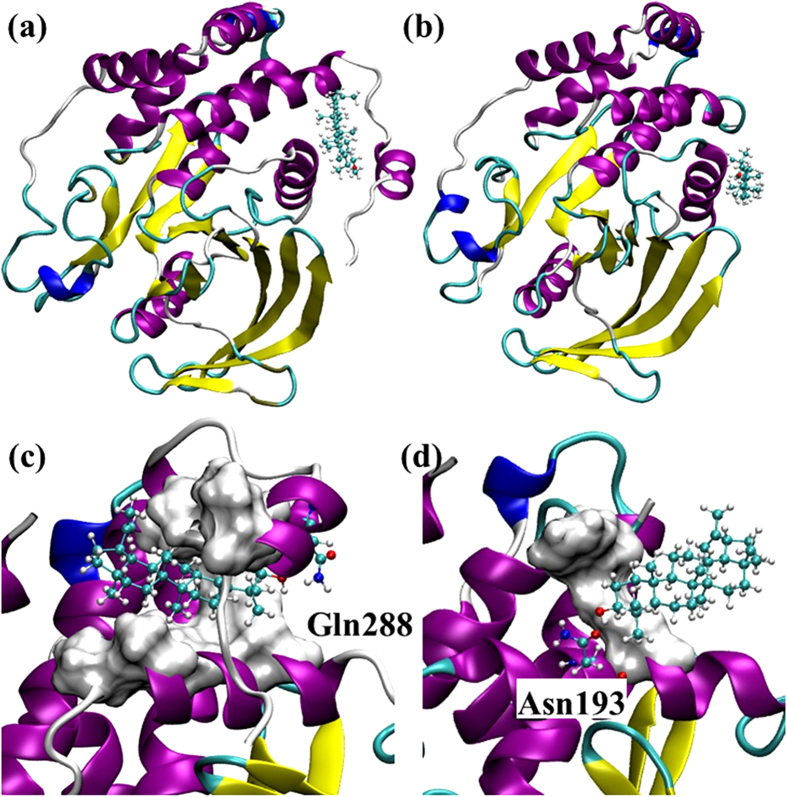
The top-ranked binding poses of lupeol to the allosteric site of PTP1B299 (**a**) and PTP1B282 (**b**). Detailed interactions revealed by the molecular dynamics simulations for lupeol and PTP1B are shown in (**c**) and (**d**). The hydrophobic residues involved in the binding in PTP1B299 (Ala189, Leu192, Phe196, Phe280, Trp291, and Leu294) are shown in Surf. The polar residue involved in the hydrogen bonding Gln288 is shown in CPK (**c**). The hydrophobic residues involved in the binding in PTP1B282 (Phe196 and Phe280) are shown in Surf while the polar residue involved in the hydrogen bonding in Asn193 is shown in CPK (**d**).

The blind docking studies revealed that lupeol preferentially binds to the identified allosteric site^16^ (Table S2 and Figure S1a). Subsequently, we obtained a more accurate picture of the binding modes from the focused docking at the allosteric site (Figure S1b). The molecular dynamics simulations of the docked complex showed a stable trajectory with the backbone positional root-mean-square deviations (RMSDs) of the PTP1B backbone atoms being 1.0 – 1.5 Å. Throughout the simulation, the WPD loop remained open, indicating that PTP1B was inactive and the timescale for the opening/closure of the WPD loop was beyond the current simulation timescale^41^. Considering that the structure of lupeol is mainly hydrophobic with only one polar hydroxyl group, it appears that hydrophobic interactions play a critical role in binding (Fig. 4).

#### α7 helix strengthens the hydrophobic interactions between lupeol and PTP1B

**Figure 5:**
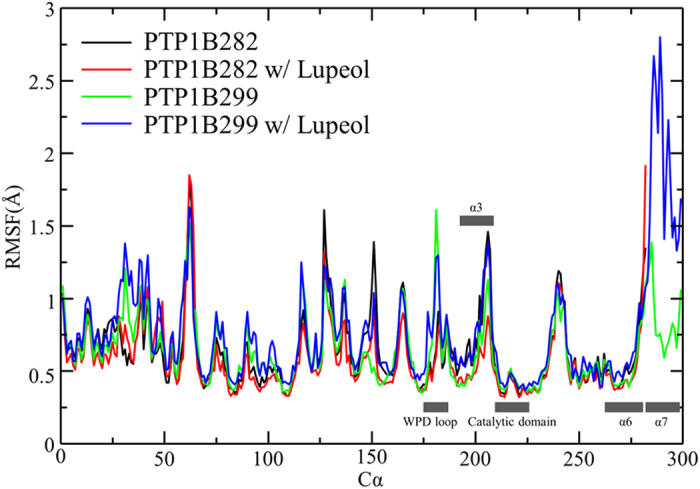
Root-mean-square fluctuations (RMSF) per residue in the equilibrium molecular dynamics simulations of PTP1B282, PTP1B282 w/Lupeol, PTP1B299, and PTP1B299 w/Lupeol. The key secondary structure elements including catalytic domain, WPD loop and allosteric site composed by α3, α6 and α7, have been labelled along X-axis.

The presence of α7 creates a hydrophobic “tunnel”, which surrounds lupeol and provides stronger hydrophobic interactions (Fig. 4c). This results in a tighter binding mode with the largest affinity of −10.3 kcal/mol (Table S3), compared to −7.7 kcal/mol (Table S4) in the PTP1B282 allosteric site. The hydrophobic tunnel comprised 6 non-polar amino acids located within 5 Å around lupeol including Ala189, Leu192, Phe196, Phe280, Trp291 and Leu294 (Fig. 4c, Table S5), which was significantly larger than the hydrophobic area formed by the two hydrophobic residues in PTP1B282 w/Lupeol (Phe196 and Phe280, Fig. 4d). We compared the solvent accessible surface areas (SASA) for PTP1B binding with lupeol (Table 1). Generally, reducing SASA increased the hydrophobic interactions. All of these six non-polar residues decreased SASAs in PTP1B299 w/Lupeol, indicating that a non-polar interaction was established between lupeol and these hydrophobic residues (Table 1). In particular, Leu192, Phe196 and Trp291 had respective 100-fold, 4-fold and 20-fold decreases in SASAs when they interacted with lupeol. These residues contributed significantly to the additional hydrophobic interactions. More importantly, individual mutations of Leu192Ala and Phe196Ala have been reported to significantly reduce the inhibitory effect of an allosteric inhibitor targeting the same binding pocket, indicating the prominent role of these residues in allosteric inhibition^42^. On the other hand, the SASAs of hydrophobic residues in PTP1B299 w/Lupeol were significantly lower than in PTP1B282 w/Lupeol (Table 1). This indicates that the existence of α7 led to a larger hydrophobic area to wrap around lupeol. In addition, we compared root-mean-square fluctuations (RMSFs) for Cα atoms in PTP1B before and after lupeol binding. Lupeol binding increased the fluctuations of α7 (Fig. 5), which may lead to the disorder of α7. Such order to disorder transition upon binding has been proposed to play an important role in allosteric inhibition^16^.

#### Orientation of lupeol in the binding site changes due to the presence of α7

Notably, although lupeol binds to the same site with or without α7, its orientation changes (Fig. 4a,b). This change is likely due to the presence of α7, which changed the preferred hydrogen bonding formation at the binding site. In PTP1B299 w/Lupeol, the residue Gln288 in α7 formed a hydrogen bond with the hydroxyl group in lupeol (Fig. 4c). However, in PTP1B282 w/Lupeol, Asn193 formed a hydrogen bond with lupeol (Fig. 4d). Nevertheless, docking simulations suggest that both hydrophobic and hydrogen bonding interactions contributed to lupeol binding.

### Lupane triterpenes are allosteric inhibitors for PTP1B

Lupane triterpenes share the same skeleton structure with minor differences in their polar functional groups (Fig. 2). The key functional group of lupane triterpenes is a pentacyclic triterpene displaying a non-polar characteristic, which interacts with non-polar residues in the allosteric site of PTP1B299. It is likely that the specific polar functional group of each lupane triterpene determines the diversity of the binding modes and leads to different binding affinities targeting PTP1B. In addition to lupeol, we also subjected lupenone, betulin, and betulinic acid to molecular docking (Figures S2, S3 and S4, Tables S2 and S3). To further elucidate the difference of binding modes within the lupane family, molecular dynamic simulations were performed on these compounds (Table S1) and the top-ranked docked complexes were confirmed to be stable over the duration of 100 ns (Figures S5 and 6).

#### PTP1B299w/Lupeol

α7 enhanced the hydrophobic interactions, and created a strong hydrogen bond with lupeol (Fig. 4c). [Fig f6]The different rotamers of Gln288 in α7 formed different hydrogen bonding patterns with lupeol, with Gln288 acting as either a hydrogen bonding donor or acceptor to strengthen the interactions with lupeol. The estimated occupancy was 73% between lupeol and Gln288 during the 100 ns simulations (Fig. 7).

#### PTP1B299 w/Lupenone

**Figure 6 f6:**
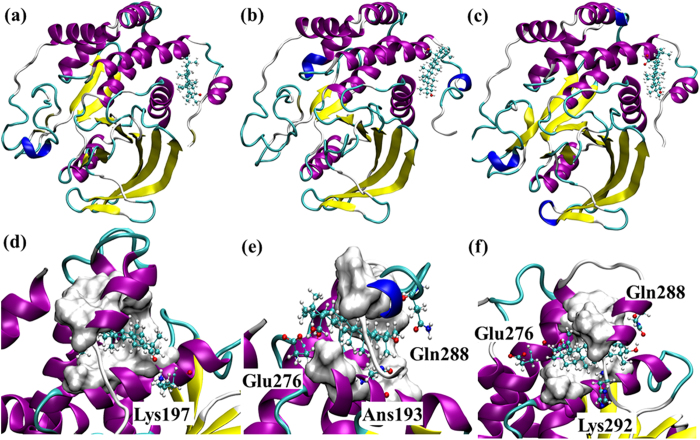
The top-ranked binding poses of lupenone (**a**), betulin (**b**), and betulinic acid (**c**) to the allosteric site of PTP1B299. The detailed interactions revealed by the molecular dynamics simulations between the ligands and PTP1B are shown in (**d**–**f**), respectively. The non-polar residues involved in the hydrophobic interactions with the ligands are shown in Surf. The polar residues involved in hydrogen bonding are shown in CPK.

**Figure 7:**
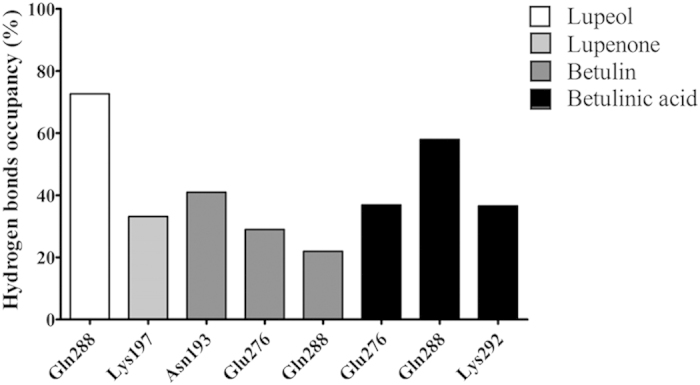
The occupancy of the hydrogen bond in the 100 ns simulations of PTP1B299 w/Lupeol, PTP1B299 w/Lupenone, PTP1B299 w/Betulin, and PTP1B299 w/Betulinic acid. A hydrogen bond is assumed to exist if the donor-acceptor distance is smaller than 3.5 Å and the donor-hydrogen-acceptor is larger than 135°. Both lupeol and lupenone perform only one hydrogen bond with PTP1B but the occupancy in lupeol is much higher than lupenone. Both betulin and betulinic acid generate multiple hydrogen bonds with PTP1B.

The polar functional group in lupenone was a carbonyl group that formed one hydrogen bond with Lys197 in the docked complex (Fig. 6d). There was a relatively low occupancy of 33% in the molecular dynamics simulations (Fig. 7). In contrast, the hydrophobic interactions formed with the non-polar residues Ala189, Leu192, Phe196, Phe280, Trp291, and Leu294 were maintained throughout the simulations (Fig. 6d, Table S5).

#### PTP1B299 w/Betulin

Betulin had two hydroxyl groups that formed multiple hydrogen bonds in the docking studies (Fig. 6e). The molecular dynamics simulations revealed the formation of three populated hydrogen bonds between betulin and Asn193, Glu276, and Gln288. The occupancies for these hydrogen bonds were 41%, 29% and 22%, respectively (Fig. 7) with considerable fluctuations in the 100 ns simulations. The hydrophobic interactions were stable and the residues involved in the binding included Ala189, Leu192, Phe196, Phe280, Trp291, and Leu294 (Fig. 6e, Table S5).

#### PTP1B299 w/Betulinic acid

Betulinic acid contained two polar functional groups that formed three stable hydrogen bonds (Fig. 6f). The carboxyl group was close to Glu276 to form one hydrogen bond with an occupancy of 37%. Additionally, betulinic acid contained a hydroxyl group, which formed another two hydrogen bonds with Gln288 (occupancy of 58%) and Lys292 (occupancy of 37%) in the 100 ns simulations (Fig. 7). Of the four lupane triterpenes, betulinic acid formed the strongest polar interactions with PTP1B. Like the other lupane triterpenes, the strong hydrophobic interactions involved in binding were with Ala189, Leu192, Phe196, Phe280, Trp291, and Leu294 (Fig. 6f, Table S5).

### Binding affinities of Lupeol and Betulinic acid to PTP1B299

The absolute binding free energies of lupeol and betulinic acid to PTP1B299 were calculated by FEP/λ-REMD. Table 2 lists the binding free energies of the two ligands examined here together with the repulsive, dispersive, electrostatic, and restrained components. The free energy of solvation is also provided. Betulinic acid had a more favourable binding than lupeol (−10.6 kcal/mol vs −12.2 kcal/mol, respectively). These binding affinities are comparable with those provided by the scoring function in AutoDock Vina (Table S3). The relative binding free energy difference of −1.6 kcal/mol compares favourably from the experimentally derived number based on IC_50_ values. Experimentally, IC_50_ is not a direct indicator of binding affinities for non-competitive inhibitors, which however could be converted to each other. According to the algorithm proposed by Cer *et al.*^43^. We estimated the constant of inhibition *k*_i_ for lupeol and betulinic acid and then calculated the binding free energies of lupeol and betulinic acid (−7.5 kcal/mol vs −8.3 kcal/mol, respectively, Table S6). The current calculations overestimate the absolute binding free energies by 3.1 kcal/mol and 3.9 kcal/mol for lupeol and betulinic acid, respectively. However, the relative binding affinity of −0.8 kcal/mol was reproduced by binding free energy calculations reasonably well (−1.6 kcal/mol). Such calculations provide confidence that in the future, relative binding free energy calculations will be applied to help the optimisation of the lead compounds with similar binding modes.

We further examined the component contributions to the binding affinities. As expected, for both ligands, the van der Waals interactions made the dominant contributions, because the allosteric site was characterised of the hydrophobic interactions with the hydrophobic skeleton of lupane triterpenes. The difference of van der Waals components between lupeol and betulinic acid to PTP1B299 was −0.6 kcal/mol (Table 2, −11.7 kcal/mol vs −12.3 kcal/mol). However, it is worth noting that betulinic acid had a slightly favourable contribution from the electrostatic interactions compared to lupeol (Table 2). The difference of electrostatic interactions in lupeol and betulinic acid was about −1.0 kcal/mol (Table 2 kcal/mol vs −0.1 kcal/mol). Such component analyses might provide useful information in future ligands optimisation process. For instance, the binding affinities might be enhanced by increasing the hydrophilicities of the groups at R_1_ and R_2_ (Fig. 2), or the compounds solubility be improved by strengthening hydrogen binding interactions by such groups. However, it is worth mentioning that one has to be cautious as such free energy components are not a state function and component analyses are pathway dependent^44^. Thus when using this information to *in silico* design new inhibitors, the (relative) binding free energy will be verified with rigorous free energy calculations.

### Lupane triterpenes selectively inhibit PTP1B activity over TCPTP

**Figure 8:**
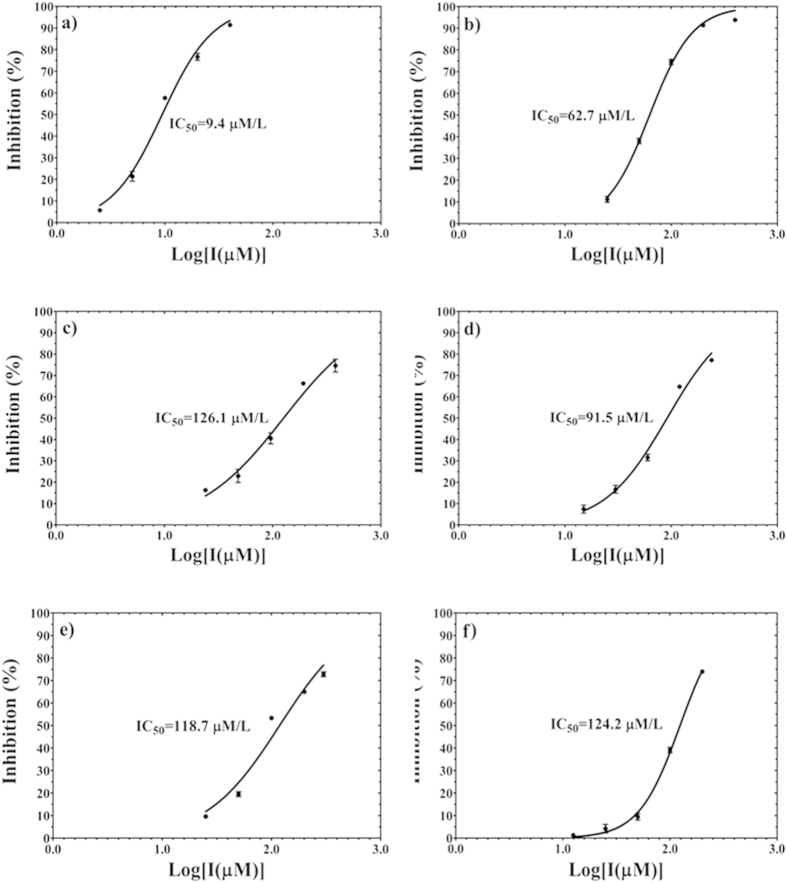
The percentage inhibition of PTP1B (**a**) and TCPTP (**b**–**f**) activity. Compound 3 is used as a control to validate the inhibition assay targeting PTP1B (**a**) and TCPTP (**b**). Four lupane triterpenes are tested for the inhibition potency of TCPTP: (**c**) Lupeol; (**d**) Lupenone; (**e**) Betulin; and (**f**) Betulinic acid.

Allosteric inhibitors are selectively characterised by the inhibition effect. We have confirmed that lupane triterpenes are PTP1B allosteric inhibitors using molecular docking and dynamics simulations. We have systematically testified the inhibitory effects of lupane triterpenes on PTP1B and TCPTP and demonstrated that lupane triterpenes specifically target PTP1B. First we applied the control drug compound 3 (Fig. 2) to both the PTP1B and TCPTP enzymes to confirm the reliability of the enzymatic assay. The IC_50_ of compound 3 on PTP1B was 9.4 μM (Fig. 8a), which was similar to that of 8.0 μM reported in the literature^16^. The IC_50_ of compound 3 on TCPTP was 62.7 μM (Fig. 8b), indicating that compound 3 had a 6.6-fold selectivity for PTP1B over TCPTP. The IC_50_ of lupane triterpenes for TCPTP was determined and compared to the IC_50_ for PTP1B as reported in the literature^21^,^22^,^23^ (Table 3). The data indicate that lupeol, lupenone, betulin and betulinic acid are modest but promising inhibitors for PTP1B with the IC_50_ being 5.6, 13.7, 15.3, and 1.5 μM, respectively. All of the lupane triterpenes had a weaker inhibitory effect on TCPTP, being 126.1 (lupeol, Fig. 8c), 91.5 (lupenone, Fig. 8d), 118.7 (betulin, Fig. 8e), and 124.2 μM (betulinic acid, Fig. 8f), respectively. In particular, lupeol and betulinic acid had a 20-fold and 80-fold selectivity for PTP1B over TCPTP, respectively.

### Lupane triterpenes inhibit PTP1B activity in an allosteric manner

**Figure 9:**
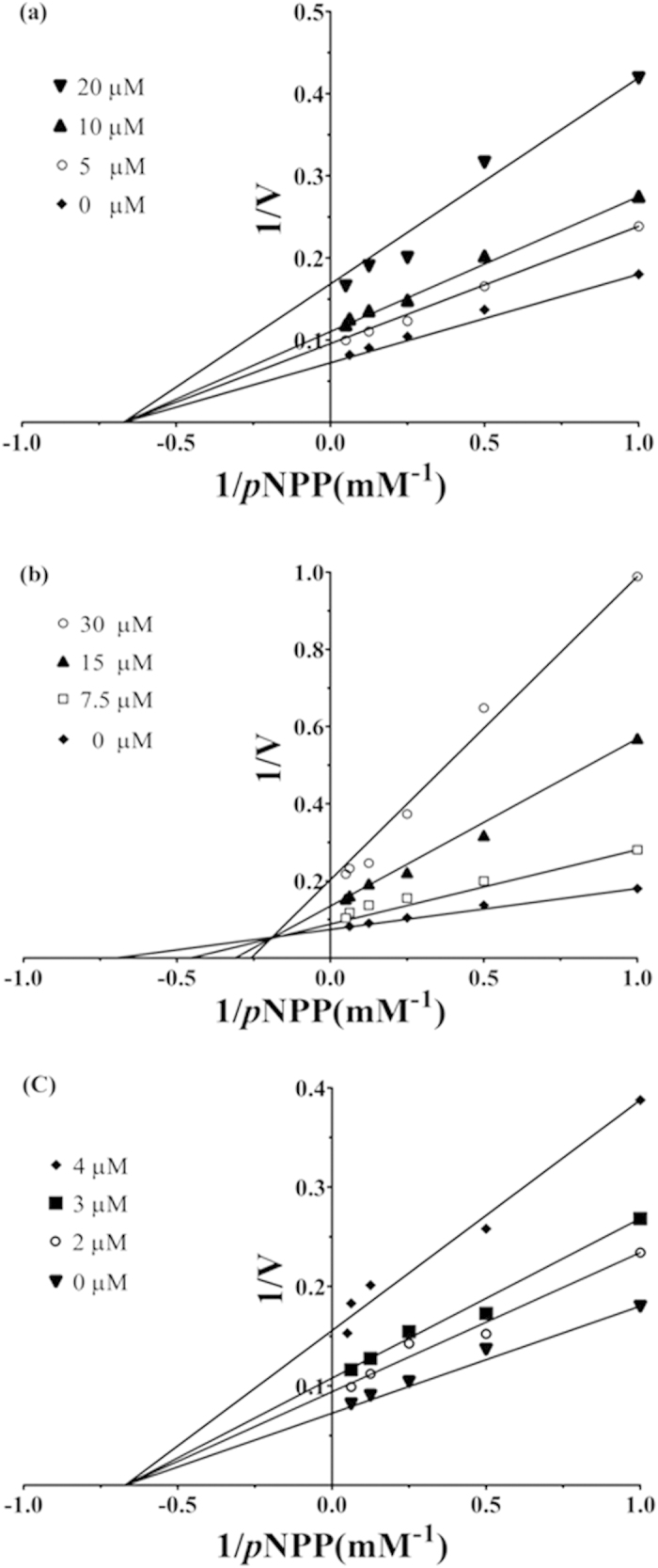
PTP1B kinetics assay of lupeol, betulin, and betulinic acid indicated by Lineweaver-Burk plot. (**a**) The concentrations of lupeol are 0 μM, 5 μM, 10 μM, and 20 μM, respectively. (**b**) The concentrations of betulin are 0 μM, 7.5 μM, 15 μM, and 30 μM, respectively. (**c**) The concentrations of betulinic acid are 0 μM, 2 μM, 3 μM, and 4 μM, respectively.

Enzyme kinetic assays have shown that lupeol and lupenone are non-competitive inhibitors for PTP1B, indicating that they target a binding site other than the active site, potentially the allosteric site^21^. We performed kinetic analysis of betulin and betulinic acid, with lupeol as a control (Fig. 9a). The modes of inhibition by betulin and betulinic acid were determined by applying the Lineweaver-Burk plot to 6 different *p*NPP and 4 different compound concentrations. As shown in Fig. 9, both betulin and betulinic acid showed a decreased *V*_max_ value, indicating that they were not competitive inhibitors. Moreover, betulinic acid was a non-competitive inhibitor having a constant *K*_m_ value with a decreased *V*_max_ value (Fig. 9b). Betulin displayed an altered *K*_m_ value and a decreased *V*_max_ value, classified as mixed-type inhibition (Fig. 9a). None of lupane triterpenes were competitive inhibitors indicating that these compounds target an allosteric binding site.

### Lupane triterpenes attenuate PTP1B expression and activity in hypothalamic neurons

#### Activation of PTP1B by TNFα in mHypoE-46 neurons

**Figure 10:**
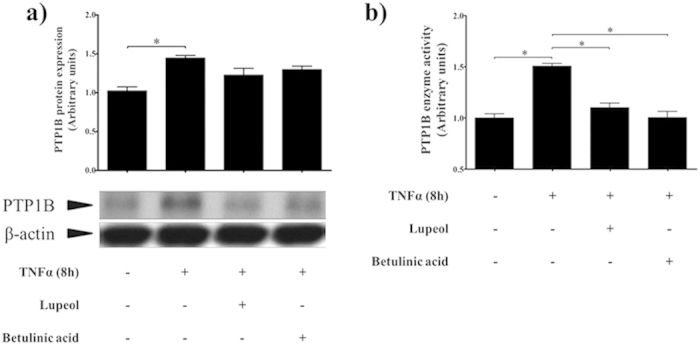
Selected lupane triterpenes inhibit PTP1B protein expression and enzyme activity in mHypoE-46 neurons. Lupane triterpenes slightly decrease TNFα-induced PTP1B protein expression (**a**), and significantly inhibit PTP1B enzyme activity (**b**). The results shown are expressed as mean ± SEM (n = 5 per group). Significance is calculated by one-way ANOVA and the *post-hoc* Tukey-Kramer HSD test. ^*^p < 0.05, versus control (without TNFα and lupane triterpenes treatments).

Neuronal PTP1B has a major role in obesity development^5^. The up-regulation of hypothalamic neuronal PTP1B expression induced by TNFα has been found in animal models^1^,^7^,^45^. In this study, we applied TNFα to induce PTP1B expression and activity in a mouse hypothalamic cell line (mHypoE-46). TNFα (20 ng/ml) had a time-course effect on PTP1B which increased PTP1B expression 1.75-fold (Figure S6), and PTP1B activity 1.5-fold (Fig. 10b) after 8 h treatment. We then investigated the cellular activity of lupane triterpenes in inhibiting PTP1B expression and activity. Lupeol and betulinic acid were chosen for the *in vitro* experiments as they showed strong potency and selectivity in inhibiting PTP1B in the enzymatic assays (Table 3).

#### Lupeol and betulinic acid inhibit cellular PTP1B activity

Lupeol (28 μM, 5× the IC_50_ value) and betulinic acid (7.5 μM, 5× the IC_50_ value) were administrated to the mHypoE-46 cell line for 10 h after treatment of TNFα for 8 h. The cells were collected for western blot and immunoprecipitation analysis. The Western blot results showed that lupeol and betulinic acid slightly decreased PTP1B protein expression (Fig. 10a). However, immunoprecipitation revealed that PTP1B enzyme activity was significantly inhibited by 35% (Fig. 10b). This indicates that lupeol and betulinic acid penetrated the cell membrane and reduced cellular PTP1B enzymatic activity.

## Discussion

There is compelling evidence that PTP1B is a promising therapeutic target for treating obesity and other diseases. However, it is a major challenge to develop a potent and selective PTP1B inhibitor. Only a few selective PTP1B inhibitors with acceptable pharmacological properties have been reported in the literature, such as TransTech Pharma Inc TTP814, and Ohr Pharmaceutical Inc Trodusquemine (MSI-1436)42, which are in Phase II and Phase I testing, respectively18. Recently, allosteric inhibition has become a promising alternative strategy to develop selective PTP1B inhibitors. The current study firstly predicts that lupane triterpenes bind to the PTP1B allosteric site by the application of molecular docking and molecular dynamics simulations. Enzymatic assays and neuronal cell cultures were used to demonstrate that lupane triterpenes are potential selective and allosteric inhibitors that target PTP1B. PTP1B allosteric inhibitor is featured by displaying a selective inhibitory effect on PTP1B over TCPTP. In an addition to these results, we also performed blind docking of lupane triterpenes targeting TCPTP (Figure S7, Table S7) and compared the results to those targeting PTP1B (Figure S1a, S2a, S3a and S4a). For each lupane triterpene, it is clear that only one pose among the top-ranked poses binds to the region which is equivalent to the allosteric site in PTP1B (Table S7). Thus the less-conserved PTP1B allosteric site is an ideal target for lupane triterpenes to inhibit PTP1B activity. This specificity may cause fewer side effects than the PTP1B active site inhibitors.

Previous structural and biochemical studies have unveiled the important role of α7 as a regulatory helix in the PTP1B conformational transition^16^. However, its mechanism remains unclear. Our results elucidate the contribution of α7 to the allosteric binding mode. Firstly, α7 is involved in the formation of PTP1B-ligand binding. The presence of α7 determines the orientation of lupeol (Fig. 4a,b) and RMSF data reveals a high fluctuation of α7 due to the existence of lupeol (Fig. 5). Therefore α7 directly interacts with lupeol to form the protein-ligand complex. Moreover, using blind and focused docking, we observed stronger binding affinities of lupeol in the presence of α7 (Table S3 and Table S4). SASA results indicate that the presence of α7 results in increased hydrophobic interactions as α7 provides more non-polar residues (Table 1). Lupeol contains a non-polar pentacyclic structure, which easily forms strong hydrophobic interactions with the hydrophobic “tunnel” formed by α7. In addition, experimental and computational studies on lupenone, betulin, and betulinic acid consolidate the role of α7.

Lupane triterpenes are of great interest to traditional medicine^46^,^47^,^48^. Lupeol has been reported to be an anti-diabetes agent^49^,^50^, an anti-cancer agent^51^, and an anti-inflammation agent^46^. Betulinic acid also shows anti-obesity activity^52^, anti-HIV activity^53^, and anti-cancer activity^53^. However, the exact target of lupane triterpenes remains unclear. Our Western blot and immunoprecipitation results show that lupeol and betulinic acid do not significantly decrease PTP1B protein expression in hypothalamic neurons (Fig. 10a). In contrast, PTP1B enzyme activity is significantly inhibited (Fig. 10b). Thus lupeol and betulinic acid directly inhibit PTP1B enzyme activity *in vitro.*

PTP1B is also involved in many diseases including cancer^54^, inflammation^7^, and diabetes^55^. Since lupane triterpenes directly target PTP1B activity, they are a potential treatment for several diseases. Betulinic acid shows higher inhibition potency as its polar functional groups form stronger hydrogen bonding interactions than lupeol, lupenone, and betulin (Fig. 7). Consistent with the IC_50_ data in table 3, the results from binding free energy calculations showed that betulinic acid had a better inhibitory effect than lupeol. Their contributions of van del waal interactions were similar, therefore the electrostatic interactions mainly contributed to the slightly lower ΔG_b_^o^ for betulinic acid (Table 2). It is encouraging to see the relative consistency between experimental results and computed data in our current work. More importantly, via investigating the structure of lupane triterpenes (Fig. 2), it is clear to notice that lupane triterpenes share a highly hydrophobic pentacyclic main structure which lead to the similarity of van del waal interactions between lupeol and betulinic acid. On the other hand, the structural difference between lupeol and betulinic acid lies in the R_2_ group, which plays a vital role strongly increasing the inhibitory effect of betulinic acid. Since the non-polar pentacyclic structure of lupane triterpenes is the premise for binding to the less polar allosteric site, modifying the polar functional group is a pivotal way to increase binding affinity and inhibition potency. R_2_ (Fig. 2) is regarded as an ideal site to be modified to introduce the polar functional groups. However, such modifications need careful investigation since this will affect their pharmacokinetic properties.

## Conclusion

As an important negative regulator in controlling human energy homeostasis, PTP1B is an attractive drug target for preventing and treating obesity and its associated metabolic syndromes. We establish computational modelling for lupane triterpenes binding to PTP1B, and demonstrate that lupane triterpenes function as allosteric inhibitors targeting PTP1B. Our future work will include detailed structural characterizations of the PTP1B-lupane triterpene complex and the rational optimization of these compounds for better efficacy. Thus exploring lupane triterpenes offers the opportunity to develop novel PTP1B allosteric inhibitors with high potency, selectivity, and few side effects.

## Additional Information

**How to cite this article**: Jin, T. *et al.* Selective binding modes and allosteric inhibitory effects of lupane triterpenes on protein tyrosine phosphatase 1B. *Sci. Rep.* **6**, 20766; doi: 10.1038/srep20766 (2016).

## Supplementary Material

Supplementary Information

## Figures and Tables

**Figure 1:**
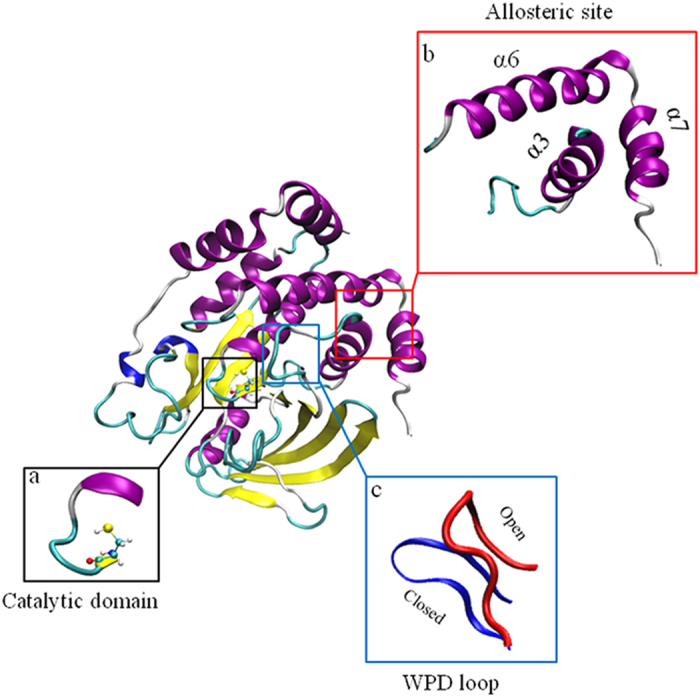
The crystallography structure of PTP1B. PTP1B has an active site Cysteine 215 with surrounding catalytic loop (**a**) and a previous identified allosteric site (**b**) which is surrounded by α3 helix, α6 helix and α7 helix. During PTP1B activation, WPD loop (**c**) moves from the open position to the closed position.

**Table 1:** Changes in solvent accessible surface area (SASA) values upon lupane triterpenes to PTP1B.

	Ala189	Leu192	Phe196	Phe280	Trp291	Leu294
PTP1B299	47.6	29.1	90.7	83.4	109.3	138.0
PTP1B299 w/Lupeol	7.9	0.3	25.9	64.2	5.5	105.1
PTP1B282 w/Lupeol	50.4	14.9	50.7	107.4		

The values are the non-polar residues in the PTP1B299, PTP1B299 w/Lupeol complex, and PTP1B282 w/Lupeol complex over the trajectories of equilibrated simulations (in Å^2^).

**Table 2:** Absolute binding free energies of lupeol and betulinic acid to PTP1B.

	ΔG_b_^o^	ΔG_total_	ΔG_rep_	ΔG_disp_	ΔG_elec_	ΔG_rstr_
Lupeol^a^		−3.8 ± 0.4	43.7 ± 0.4	−42.1 ± 0.2	−5.4 ± 0.1	
Betulinic acid^a^		−9.2 ± 0.4	44.5 ± 0.3	−43.3 ± 0.2	−10.4 ± 0.3	
Lupeol−PTP1B299^b^	−10.6 ± 0.6	−14.4 ± 0.6	54.9 ± 0.5	−65.0 ± 0.2	−4.5 ± 0.2	0.2
Betulinic acid-PTP1B299^b^	−12.2 ± 0.7	−21.4 ± 0.7	56.0 ± 0.6	−67.1 ± 0.2	−10.5 ± 0.2	0.2

The repulsive (ΔG_rep_), dispersive (ΔG_disp_), electrostatic (ΔG_elec_) and restraining (ΔG_rstr_) contributions sum up to the total in the FEP/λ-REMD simulations^31^,^32^. The error is estimated on the four sequential simulations with each of 0.2 ns for each replica. There are two separate free energy calculations, where in (a) a solvated ligand is decoupled from the aqueous solution to vacuo, and in (b) a bound ligand is decoupled from the enzyme in solution. The difference between values of (a) and (b) is the absolute binding free energy for a ligand to PTP1B (ΔG_b_^o^).

**Table 3:** Inhibition potency of lupane triterpenes (IC_50_) targeting PTP1B and TCPTP.

	PTP1B (μM)	TCPTP (μM)	Fold changes
Lupeol	5.6^21^	126.1	22
Lupenone	13.7^21^	91.5	6
Betulin	15.3^23^	118.7	7
Betulinic acid	1.5^22^	124.2	81

The data for PTP1B are taken from refs 21, 22, 23.
