# Supplementary material for: Selective binding modes and allosteric inhibitory effects of lupane triterpenes on protein tyrosine phosphatase 1B
Source: Sci Rep. 2016 Feb 11;6:20766. doi: 10.1038/srep20766 (PMC4749975; doi:10.1038/srep20766)
Supplement: Supplementary Information [file srep20766-s1.pdf]

Selective binding modes and allosteric inhibitory effects of lupane triterpenes on protein tyrosine phosphatase 1B

Tiantian Jin [a], Haibo Yu [b *] and Xu-Feng Huang [a *]

[a] Centre for Translational Neuroscience, School of Medicine, University of Wollongong, and Illawarra Health and Medical Research Institute (IHMRI), Wollongong, NSW 2522, Australia
[b] School of Chemistry, University of Wollongong, Wollongong, NSW 2522, Australia
[*] Corresponding Author: xhuang@uow.edu.au; hyu@uow.edu.au

Table S1: Summary of the simulated systems.

Label	PTP1B282	PTP1B282 w/ Compound2	PTP1B282 w/ Lupeol	PTP1B299	PTP1B299 w/ Compound2	PTP1B299 w/ Lupeol	PTP1B299 w/ Lupenone	PTP1B299 w/ Betulin	PTP1B299 w/ Betulinic acid
PTP1B[a]	282	282	282	299	299	299	299	299	299
Ligand	-	Compound 2	Lupeol	-	Compound 2	Lupeol	Lupenone	Betulin	Betulinic acid
Time (ns)	20	20	20	20	100	100	100	100	100

a: the number of residues included in the simulations.

Table S2. List of poses in blind docking of lupane triterpenes targeting PTP1B299. Only those top 20 binding modes with binding affinities less than 3.0 kcal/mol and higher than the best binding mode are listed. # indicates that the ligand binds to the proposed allosteric site.

	Binding Affinities (kcal/mol)			
Poses	Lupeol	Lupenone	Betulin	Betulinic acid
1	-10.3#	-10.4#	-10.7#	-10.1#
2	-10.1#	-10.1#	-9.9#	-10.0#
3	-9.1#	-8.9#	-9.1#	-8.7#
4	-8.2#	-7.9#	-8.5#	-7.2
5	-7.3	-7.8#	-7.9#	-7.2
6	-7.3#	-7.5	-7.7	-7.0#
7	-7.1	-7.5#	-7.4#	-7.0#
8	-7.0	-7.3#	-7.3#	-6.9
9	-7.0	-7.1	-7.3	-6.8#
10	-6.9#	-7.0	-7.3#	-6.8
11	-6.9	-7.0#	-7.2	-6.8
12	-6.9	-6.9	-7.2	-6.8
13	-6.8	-6.9	-7.2	-6.7
14	-6.8	-6.9	-7.1	-6.7
15	-6.8#	-6.9	-7.1	-6.7
16	-6.8	-6.8	-7.1	-6.7
17	-6.7	-6.8#	-7.1	-6.7
18	-6.7	-6.7	-7.1	-6.7
19	-6.7#	-6.7	-7.1	-6.6
20	-6.6		-7.0	-6.6

Table S3. List of poses in the focused docking of lupane triterpenes targeting PTP1B299. Only those binding modes with binding affinities less than 3.0 kcal/mol and higher than the best binding mode are listed. The highest-ranking pose was subjected to further molecular dynamics simulations. These data indicate that the default scoring function in AutoDock Vina can not differentiate the four lupane triterpenes, i.e. which is a more potent inhibitor.

	Binding Affinities (kcal/mol)			
Poses	Lupeol	Lupenone	Betulin	Betulinic acid
1	-10.3	-10.4	-10.7	-10.1
2	-10.1	-10.1	-10.0	-10.0
3	-9.1	-8.9	-9.8	-8.7
4	-7.2		-9.0	-8.6
5	-6.9		-7.3	-7.0

Table S4. List of poses in the focused docking of lupane triterpenes targeting PTP1B282. Only those binding modes with binding affinities less than 3.0 kcal/mol and higher than the best binding mode are listed.

	Binding Affinities (kcal/mol)			
Poses	Lupeol	Lupenone	Betulin	Betulinic acid
1	-7.7	-7.8	-7.1	-7.5
2	-7.2	-7.0	-6.8	-7.0
3	-7.1	-6.9	-6.8	-6.8
4	-6.8	-6.9	-6.6	-6.4
5	-6.7	-6.8	-6.2	-6.3

Table S5. Average minimal distances between lupane triterpenes and non-polar residues in the allosteric binding site in PTP1B.

	Distance (Å)			
	Lupeol	Lupenone	Betulin	Betulinic acid
Ala189	4.1	3.8	4.2	3.9
Leu192	3.9	3.9	4.0	4.0
Phe196	3.7	3.7	3.7	3.6
Phe280	3.5	3.6	3.7	3.7
Trp291	3.7	3.7	3.7	3.6
Leu294	4.1	4.1	5.0	4.1

The values are the average minimal distance between the heavy atoms in non-polar residues and ligands in the PTP1B299 w/ Lupeol complex, PTP1B299 w/ Lupenone complex, PTP1B299 w/ Betulin complex, and PTP1B299 w/ Betulinic acid complex for the trajectory of 100 ns.

Table S6. Estimation of binging free energy based on experimental IC50 values.

	Parameters (μM)					ΔG_b^o (kcal/mol)
	Enzyme concentration [E]	Substrate concentration [S]	Michaelis-Menten constant [K_m]	concentration for 50% inhibition [IC_{50}]	Constant of inhibition [K_i]	
Lupeol	0.01	20000	1.54	5.6[1]	5.6	-7.5
Lupenone	N/A	N/A	N/A	13.7[1]	N/A	N/A
Betulin	0.01	20000	2.8	15.3[2]	15.3	-6.8
Betulinic acid	0.01	20000	1.47	1.5[3]	1.5	-8.3

The K_i values are calculated via the online IC50-to-Ki converter tool (http://botdb.abcc.ncifcrf.gov/toxin/kiCalES.jsp). According to the equation, for non-competitive and Uncompetitive inhibitors, When [S] >> K_m, $K_i = IC_{50}$.

Table S7. List of poses in the blind docking of lupane triterpenes targeting TCPTP. Only those top 20 binding modes with binding affinities less than 3.0 kcal/mol and higher than the best binding mode are listed. * indicates that the ligand binds to the active site. # indicates that the ligand binds to the region which is equivalent to the allosteric site in PTP1B.

	Binding Affinities (kcal/mol)			
Poses	Lupeol	Lupenone	Betulin	Betulinic acid
1	-8.2	-8.5	-7.9	-8.0
2	-8.0	-8.0#	-7.4	-7.7
3	-8.0	-8.0	-7.4	-7.5
4	-7.9	-8.0	-7.4	-7.5
5	-7.9	-7.9	-7.4	-7.4
6	-7.7	-7.8	-7.3	-7.4
7	-7.7#	-7.7*	-7.3	-7.3
8	-7.6	-7.7	-7.2	-7.3
9	-7.6	-7.7	-7.1	-7.2
10	-7.5*	-7.7	-7.1	-7.2
11	-7.5	-7.5	-7.0#	-7.2#
12	-7.5	-7.5	-7.0	-7.1
13	-7.4	-7.5	-7.0	-7.1
14	-7.3	-7.5*	-7.0	-7.1
15	-7.3*	-7.5	-7.0	-7.1
16	-7.2	-7.4	-6.9*	-7.0
17	-7.2	-7.4	-6.8	-7.0*
18	-7.2	-7.3	-6.8	-7.0*
19	-7.2	-7.3	-6.8	-7.0
20	-7.1	-7.2*	-6.8*	

Figure S1. Docking poses of lupeol binding to PTP1B299. (a) Top 20 blind docking poses are listed. Poses at the proposed allosteric sites are shown in different colours with the rest in cyan. There are no poses identified which bind to the active site. (b) Top 5 focused docking poses at the proposed allosteric site (listed in Table S1).

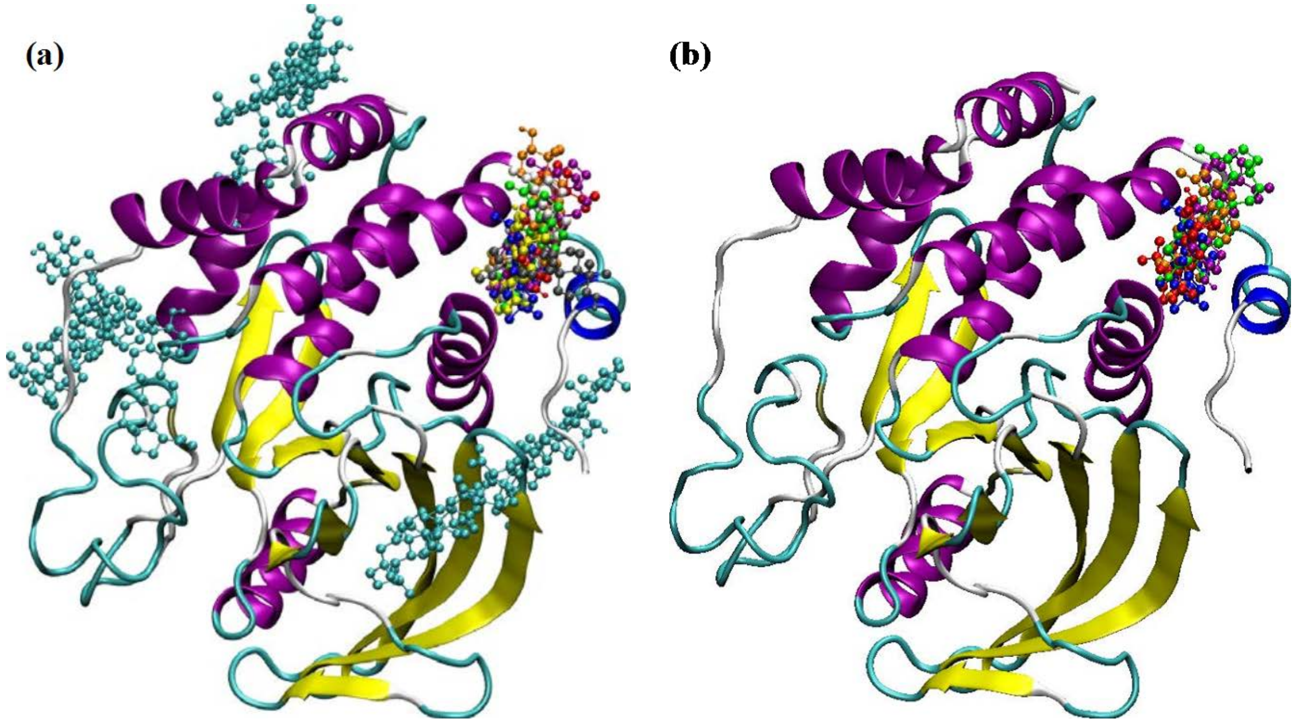

Figure S2. Docking poses of lupenone binding to PTP1B299. (a) Top 19 blind docking poses are listed. Poses at the proposed allosteric sites are shown in different colours with the rest in cyan. There are no poses identified which bind to the active site. (b) Top 3 focused docking poses at the proposed allosteric site (listed in Table S1).

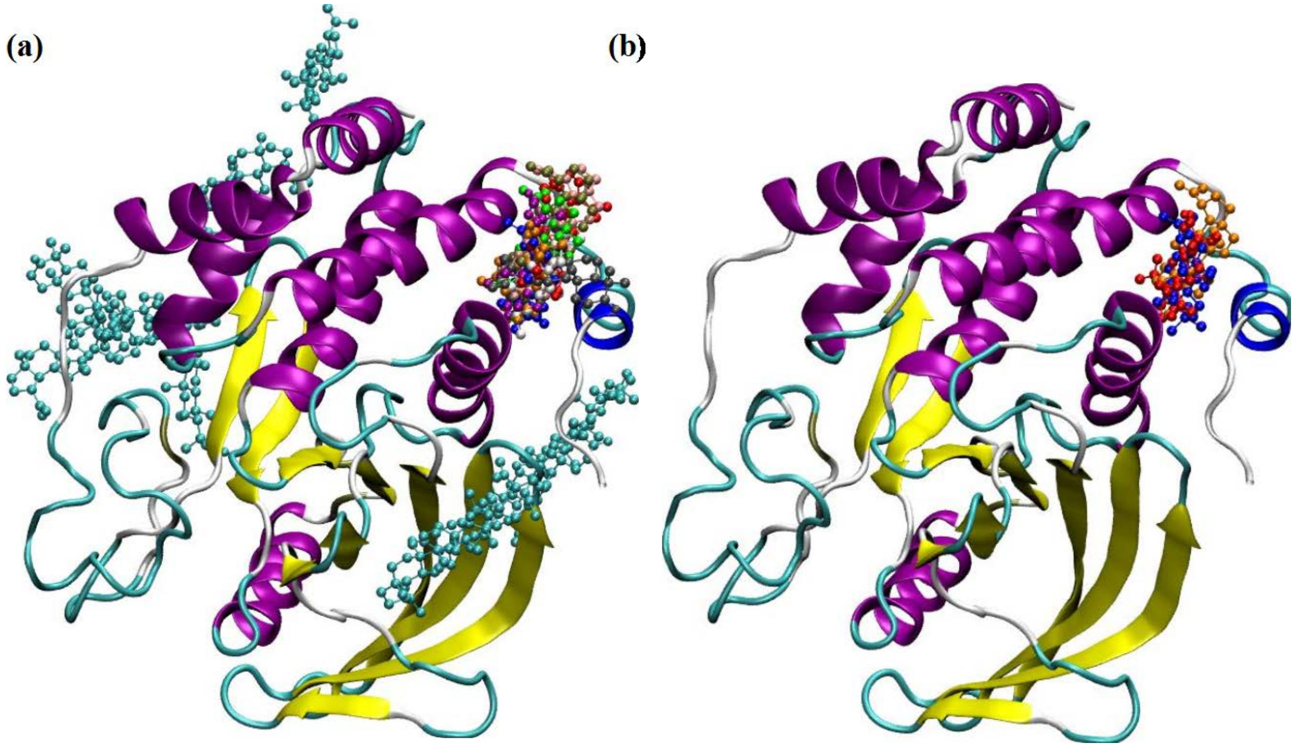

Figure S3. Docking poses of betulin binding to PTP1B299. (a) Top 20 blind docking poses are listed. Poses at the proposed allosteric sites are shown in different colours with the rest in cyan. There are no poses identified which bind to the active site. (b) Top 5 focused docking poses at the proposed allosteric site (listed in Table S1).

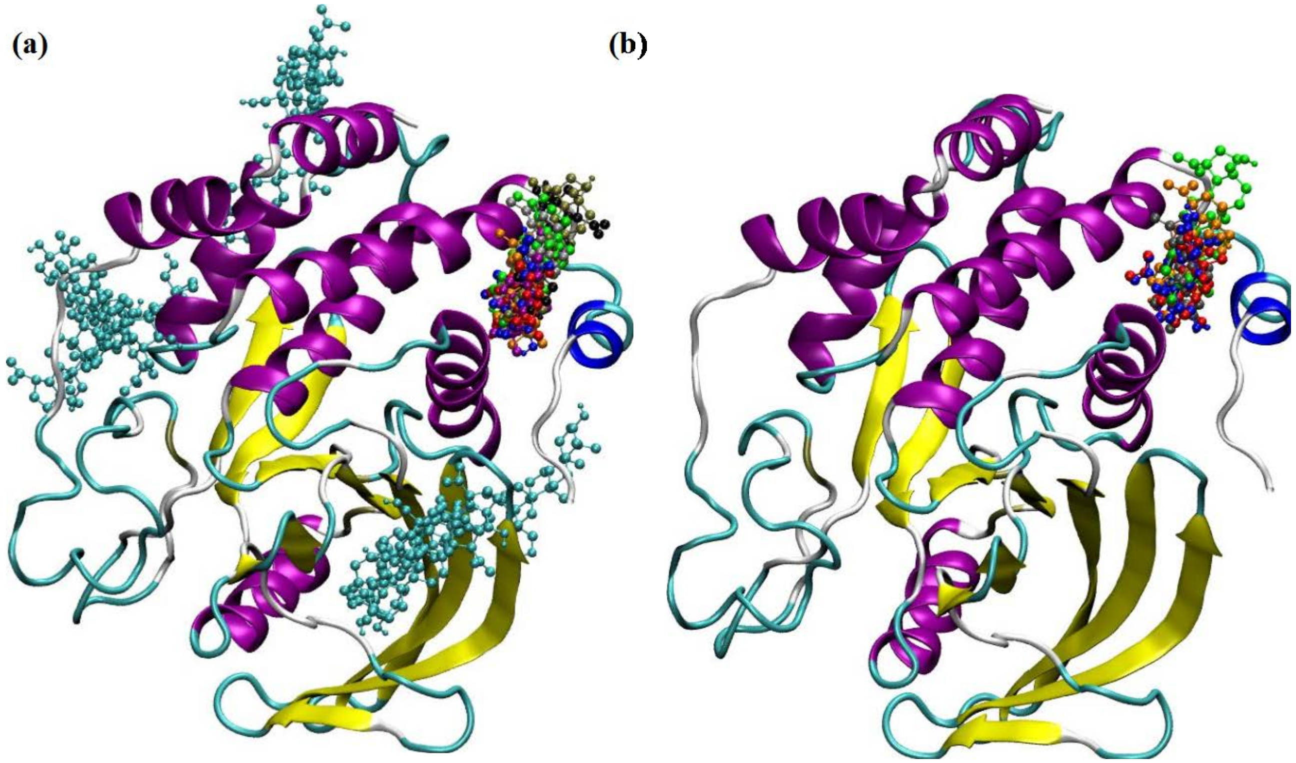

Figure S4. Docking poses of betulinic acid binding to PTP1B299. (a) Top 20 blind docking poses are listed. Poses at the proposed allosteric sites are shown in different colours with the rest in cyan. There are no poses identified which bind to the active site. (b) Top 5 focused docking poses at the proposed allosteric site (listed in Table S1).

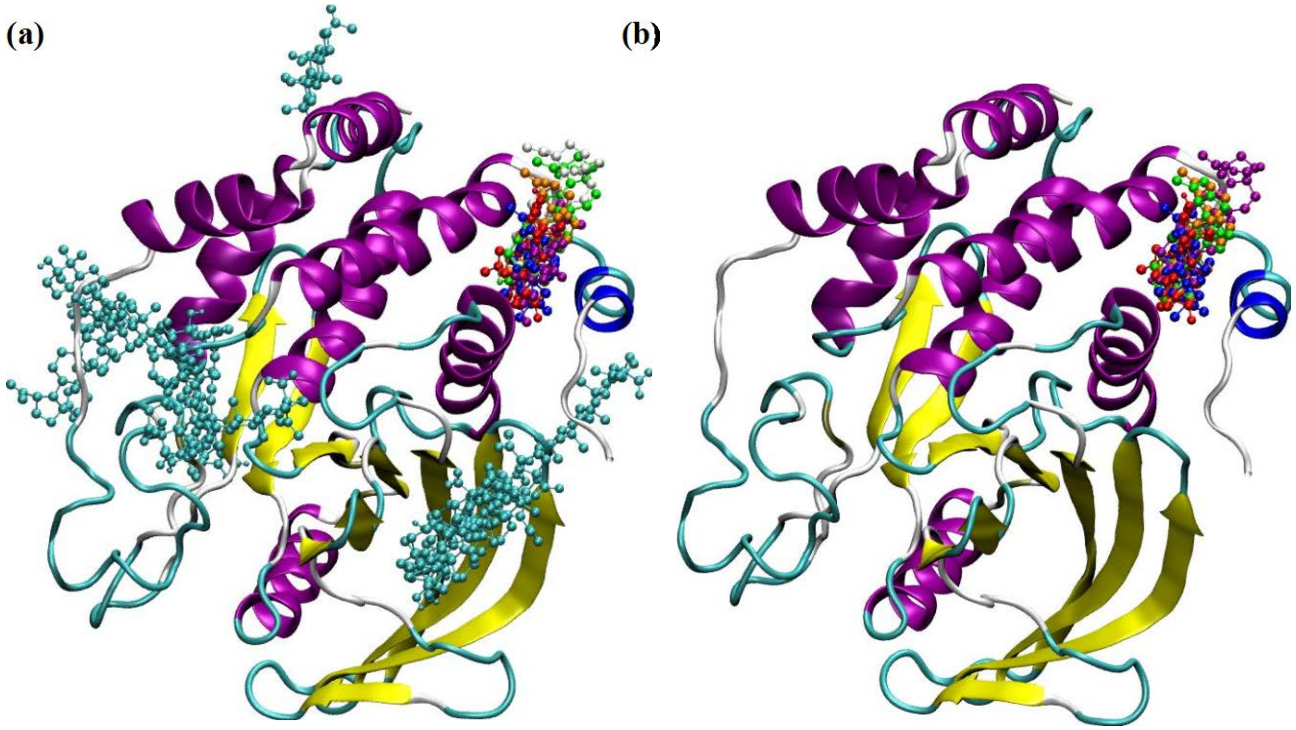

Figure S5. Backbone root-mean-square deviations (RMSD) in the 100 ns molecular dynamics simulations of PTP1B299 w/ Lupeol, PTP1B299 w/ Lupenone, PTP1B299 w/ Betulin, and PTP1B299 w/ Betulinic acid.

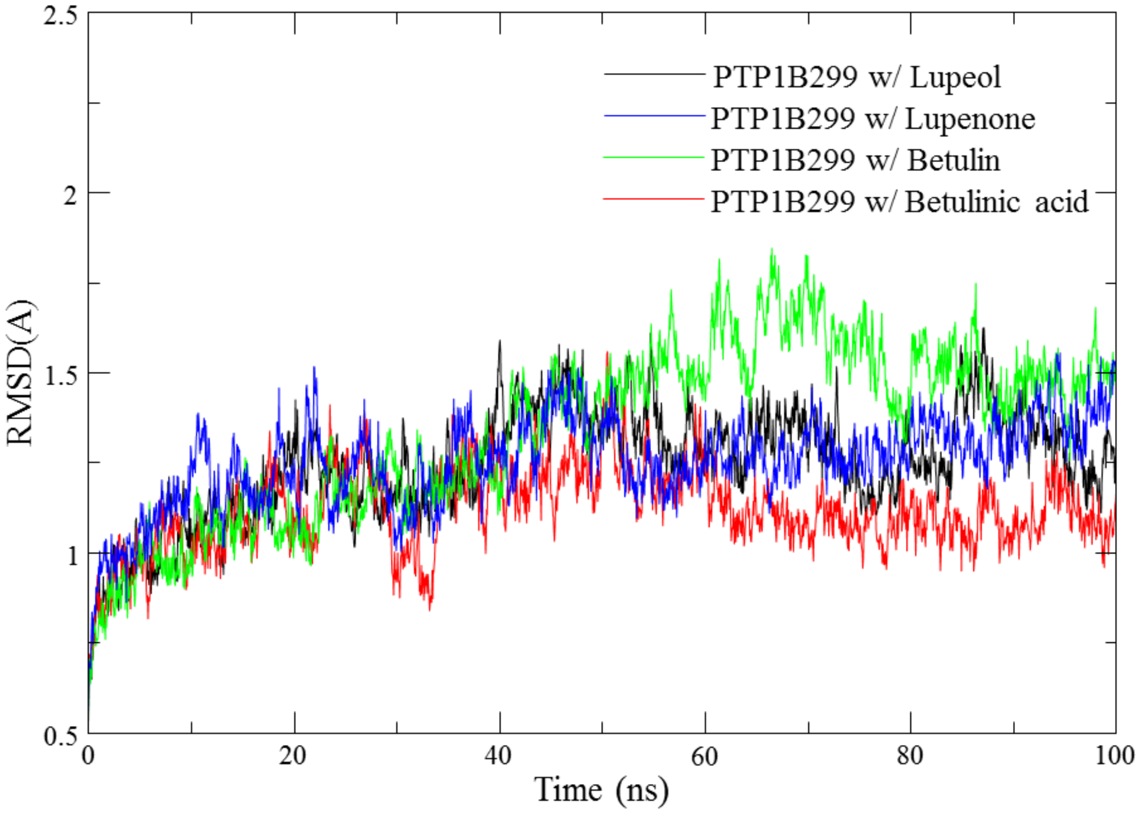

Figure S6. Time-course effects of 20ng/ml TNFα on PTP1B expression in mHypoE-46 neurons. The results shown are expressed as mean ± SEM (n=5 per group). Significance was calculated by one-way ANOVA and the *post-hoc* Tukey-Kramer HSD test. * $p < 0.05$, ** $p < 0.01$ versus control (Without TNFα stimulation).

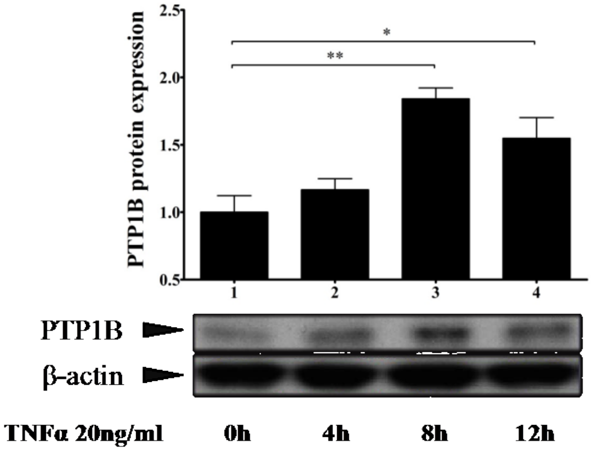

Figure S7. Blind docking poses of lupane triterpenes binding to TCPTP. Only those top 20 binding modes with binding affinities less than 3.0 kcal/mol and higher than the best binding mode are listed. (a) lupeol; (b) lupenone; (c) betulin; (d) betulinic acid. Those poses at the active sites and the region which is equivalent to the allosteric site of PTP1B are shown in different colours with the rest in cyan.

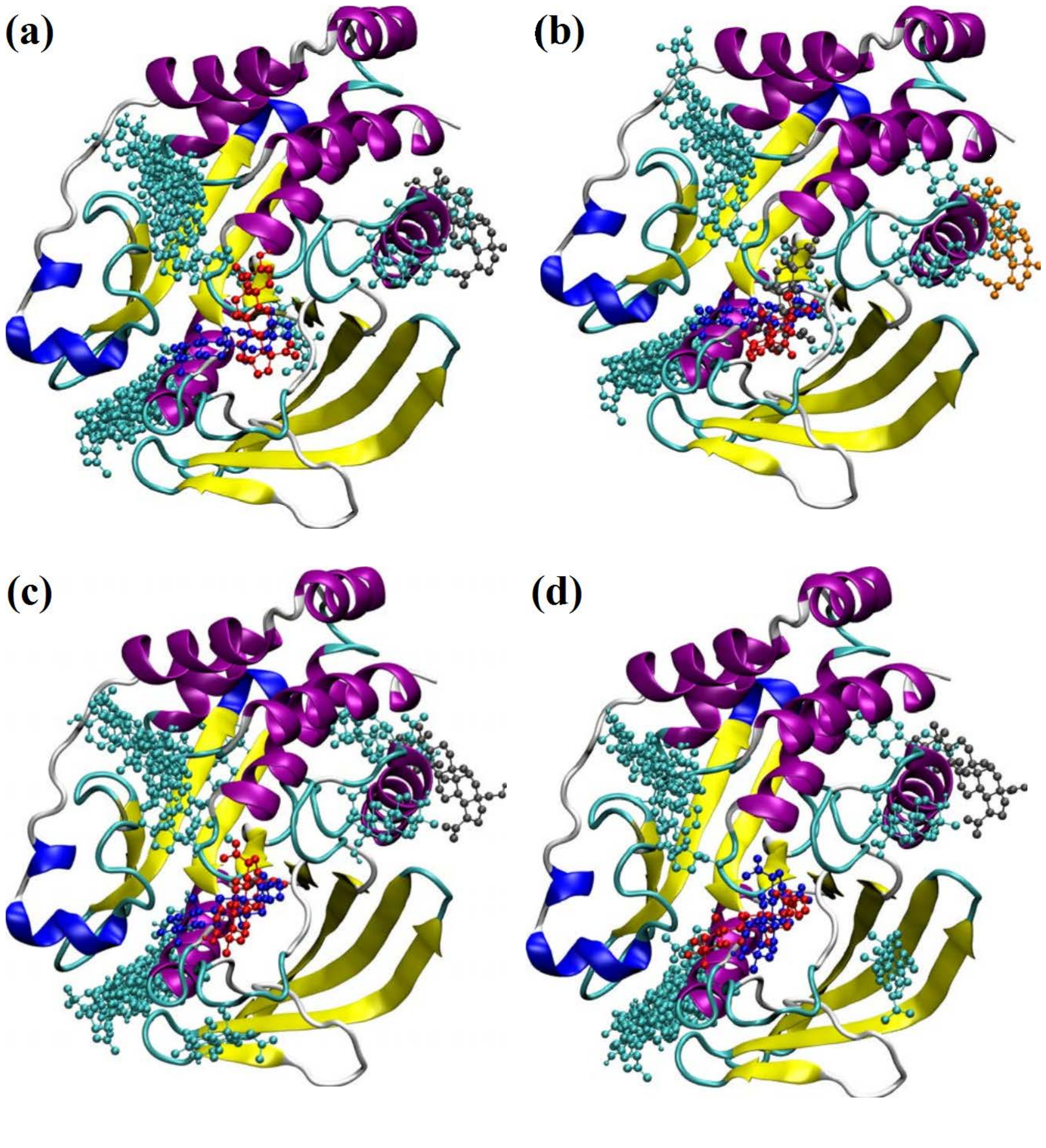

Reference:

1 Na, M., Kim, B. Y., Osada, H. & Ahn, J. S. Inhibition of protein tyrosine phosphatase 1B by lupeol and lupenone isolated from Sorbus commixta. *J Enzyme Inhib Med Chem* **24**, 1056-1059, doi:10.1080/14756360802693312 (2009).

2 Xu, W. *et al.* Chemical Constituents of the Roots of Euphorbia micractina. *J Nat Prod* **72**, 1620-1626, doi:10.1021/np900305j (2009).

3 Choi, J. Y. *et al.* Isolation of betulinic acid, its methyl ester and guaiane sesquiterpenoids with protein tyrosine phosphatase 1B inhibitory activity from the roots of Saussurea lappa C.B.Clarke. *Molecules* **14**, 266-272, doi:10.3390/molecules14010266 (2009).
